# Genomic analyses of *Burkholderia cenocepacia* reveal multiple species with differential host-adaptation to plants and humans

**DOI:** 10.1186/s12864-019-6186-z

**Published:** 2019-11-04

**Authors:** Adrian Wallner, Eoghan King, Eddy L. M. Ngonkeu, Lionel Moulin, Gilles Béna

**Affiliations:** 1 IRD, CIRAD, University of Montpellier, IPME; 911 avenue Agropolis, BP 64501, 34394 Montpellier, France; 2 Institute of Agronomic Research for Development (IRAD), PO Box 2123, Yaoundé, Cameroon

**Keywords:** *Burkholderia cenocepacia*, Opportunistic pathogen, Comparative genomics, Host adaptation, PGPR

## Abstract

**Background:**

*Burkholderia cenocepacia* is a human opportunistic pathogen causing devastating symptoms in patients suffering from immunodeficiency and cystic fibrosis. Out of the 303 *B. cenocepacia* strains with available genomes, the large majority were isolated from a clinical context. However, several isolates originate from other environmental sources ranging from aerosols to plant endosphere. Plants can represent reservoirs for human infections as some pathogens can survive and sometimes proliferate in the rhizosphere. We therefore investigated if *B. cenocepacia* had the same potential.

**Results:**

We selected genome sequences from 31 different strains, representative of the diversity of ecological niches of *B. cenocepacia*, and conducted comparative genomic analyses in the aim of finding specific niche or host-related genetic determinants. Phylogenetic analyses and whole genome average nucleotide identity suggest that strains, registered as *B. cenocepacia*, belong to at least two different species. Core-genome analyses show that the clade enriched in environmental isolates lacks multiple key virulence factors, which are conserved in the sister clade where most clinical isolates fall, including the highly virulent ET12 lineage. Similarly, several plant associated genes display an opposite distribution between the two clades. Finally, we suggest that *B. cenocepacia* underwent a host jump from plants/environment to animals, as supported by the phylogenetic analysis. We eventually propose a name for the new species that lacks several genetic traits involved in human virulence.

**Conclusion:**

Regardless of the method used, our studies resulted in a disunited perspective of the *B. cenocepacia* species. Strains currently affiliated to this taxon belong to at least two distinct species, one having lost several determining animal virulence factors.

## Background

Over the past years, the genus *Burkholderia* has been progressively revised, leading to the description of six current genera, *Burkholderia* *sensu stricto, Paraburkholderia, Caballeronia, Trinickia, Mycetohabitans* and *Robbsia* [1, 2]. *Burkholderia* sensu stricto englobes at least 31 distinct species, including 22 that belong to the *Burkholderia cepacia* complex (BCC) [1]. The BCC harbors species that are opportunistic human pathogens, causing devastating symptoms in immunocompromised individuals. These pathogens are mainly causing nosocomial infections and severely affect patients suffering from cystic fibrosis (CF). In some cases, the infected patients can develop the fatal “cepacia syndrome” characterized by progressive respiratory failure and necrotizing pneumonia, often resulting in early death [3]. However, some BCC strains seem to be more virulent than others as most infections are caused by either *Burkholderia cenocepacia* or *Burkholderia multivorans* [4]. In some regions of Europe as well as Canada, *B. cenocepacia* infections account for over 80% of bacterial infections in CF patients [5–7]. One lineage in particular, ET 12, is highly transmissible and responsible for most *B. cenocepacia* outbreaks [8]. It is no surprise that this deadly species has received a considerable amount of attention considering its clinical implication in human health [9].

The specific description of *B. cenocepacia* occurred in 2003. It was originally part of *Burkholderia cepacia* whose type strain, LMG1222, was isolated from decaying onions and identified as a plant pathogen [10]. *B. cepacia* later appeared to be recurrently isolated from immunocompromised patients and was recognized as an opportunistic pathogen. However, *B. cepacia* also proved to be useful as a biocontrol agent against plant pathogens, inhibiting growth of diverse oomycetes, fungi, bacteria and nematodes [11, 12]. With the advancements of genomics, it was demonstrated that the presumed *B. cepacia* species should be divided in five genetically distinct but phenotypically undistinguishable genomovars [13]. With further studies, the number of *B. cepacia* genomovars increased and were progressively classified into nine separate taxa, mostly using *recA*-based identification [14–16].

*B. cenocepacia* (initially genomovar III) was distinguished from *B. cepacia* by DNA-DNA hybridization studies but *recA* sequence phylogeny still suggested different subgroups within *B. cenocepacia* [17]. At least four different *recA*-lineages (IIIA, IIIB, IIIC and IIID) are observed with lineages IIIA and IIIB being predominant in clinical isolations. The highly virulent strains of the ET 12 lineage belong to group IIIA [17–19]. Moreover, using microarray experiments, it was observed that various *B. cenocepacia* strains reacted differentially to conditions mimicking the human host environment. Out of several hundreds of differentially regulated genes, only nine displayed similar regulations across the different strains, suggesting important differences in infection capacity across strains of *B. cenocepacia* [20–22]. Despite the apparent genetic contrast between *B. cenocepacia* strains, no large scale comparative genomics study has been conducted on this species yet [23].

Albeit it has received most its attention from clinical studies, it is not uncommon to recover *B. cenocepacia* isolates from soil samples. Isolates of this species have also been frequently sampled from plant material [19, 24–26]. Plants could thus represent alternative hosts and potential reservoirs for BCC strains. Still, their adaptation for plant infection or colonization remains poorly documented. Four studies investigated the biocontrol potential of recognized *B. cenocepacia* strains that all belong to the IIIB *recA*-lineage. Altogether, they suggest strong biocontrol potential of plant-associated *B. cenocepacia* strains against diverse plant-pathogens [26–29].

Our study aims at clarifying the taxonomic position of *B. cenocepacia* strains isolated from different sources by investigating the correlation between genomic identity and environmental distribution within the species. We also strive to elucidate if plants may represent a reservoir of human opportunistic *B. cenocepacia* strains. By using bioinformatics and phylogenetic tools, we compared the whole genome sequences of 31 *B. cenocepacia* strains isolated from either clinical or environmental sources. We highlight the existence of a new *Burkholderia* species and describe its reduced adaptation to animal infection and virulence as compared to its closest parent, *B. cenocepacia*.

## Results

### Characteristics of selected *B. cenocepacia* strains selected for comparative analyses

Two hundred forty-six of the 303 genomes (either full or draft) of *B. cenocepacia* strains available on the NCBI database, at the time of this study, are clinical isolates (Additional file 1: Figure S1). They were sampled from patients suffering from CF, from other pathologies or from healthy patients. The isolates also vary according to the source of biological sample they originate from. Most clinical isolates were obtained from sputum or blood samples, but some were also isolated from hospital equipment [30] as *B. cenocepacia* is resistant to many common antibiotics as well as several sanitizers [31, 32].

The remaining *B. cenocepacia* strains with available genomes come from environmental sources. These can be aerosol and water samples but also agricultural soil and plant roots (Additional file 1: Figure S1, Table 1). The *recA* phylogeny of all genomes available showed that the *recA*-IIIA lineage includes in a very large majority strains isolated from a clinical context (228 isolates; 94.2%), with only 10 strains obtained from an environmental context and four with an unknown origin (Additional file 1: Figure S1). Conversely the *recA*-IIIB clade mixed clinical isolates (18; 43.9%), environmental isolates (15; 36.6%), plant isolates (4; 9.8%) and isolates with unknown origin (4; 9.8%). It should however be noted that, among the 228 isolates clinical isolates of the *recA-*IIIA clade, 188 were isolated from the same place, a hospital in Vancouver, Canada. Similarly, 12 of the 18 clinical isolates of the *recA*-IIIB clade are from the same hospital. There is thus a strong bias in the sampling, and highly similar strains might coexist in the database.

**Table 1:** Key information on the 31 *B. cenocepacia strains* used in the phylogenetic analysis

Strain	Isolation source^a^	Localization	Affiliation^b^	Reference
842	Human nasal scrub	Malaysia	*B. cenocepacia*	Unpublished
895	Human cord blood	Malaysia	*B. cenocepacia*	Unpublished
BC-3	Human blood	India	*B. cenocepacia*	[33]
BC-7	CF patient sputum	Canada, Toronto	*B. cenocepacia*	[34]
F01	Soil	Burkina Faso	*B. cenocepacia*	[23]
GIMC4560Bcn122	Human sputum	Russia, Moscow	*B. cenocepacia*	[35]
H111	CF patient sputum	Germany	*B. cenocepacia*	[36]
J2315 T	CF patient	UK, Edinburgh	*B. cenocepacia*	[37]
K56-2Valvano	CF patient sputum	Canada, Toronto	*B. cenocepacia*	[38]
MSMSB384	Water	Australia	*B. cenocepacia*	[39]
ST32	Human sputum	Czech Republic	*B. cenocepacia*	[40]
VC1254	Human sputum	Canada, Vancouver	*B. cenocepacia*	[41]
VC2307	Human sputum	Canada, Vancouver	*B. cenocepacia*	[41]
VC12308	Human sputum	Canada, Vancouver	*B. cenocepacia*	[41]
ABIP444	Rice rhizosphere	Cameroun	*Burkholderia* sp. nov.	This study
AU1054	CF patient blood	USA	*Burkholderia* sp. nov.	[42]
CR318	Maize rhizosphere	Canada, Ontario	*Burkholderia* sp. nov.	[25]
FL-5-3-30-S1-D7	Soil	USA, Florida	*Burkholderia* sp. nov.	[43]
HI2424	Agricultural soil	USA, New York	*Burkholderia* sp. nov.	[42]
KC-01	Coastal saline soil	Bangladesh	*Burkholderia* sp. nov.	[44]
MC0–3	Maize rhizosphere	USA, Michigan	*Burkholderia* sp. nov.	[19]
PC184Mulks	Human sputum	USA, Ohio	*Burkholderia* sp. nov.	Unpublished
Tatl-371	Tomato rhizosphere	Mexico, Morelos	*Burkholderia* sp. nov.	[26]
VC7848	Human sputum	Canada, Vancouver	*Burkholderia* sp. nov.	[41]
VC12802	Human sputum	Canada, Vancouver	*Burkholderia* sp. nov.	[41]
Bp8974	Soil	Puerto RIco	Undefined species	Unpublished
Bp9038	Water	Puerto RIco	Undefined species	Unpublished
CEIB S5–2	Agricultural soil	Mexico, Tepoztlan	Undefined species	[45]
DWS 37E-2	Soil	Australia	*B. latens*	[46]
DDS 22E-1	Aerosol	Australia	*B. pseudomultivorans*	[46]
869 T2	Vetiver endophyte	Taiwan	*B. seminalis*	[47]

^a^For human isolates, the patient’s condition is specified when known

^b^Based on the information acquired during this study

### Core-genome phylogenetic analysis

**Fig. 1:**
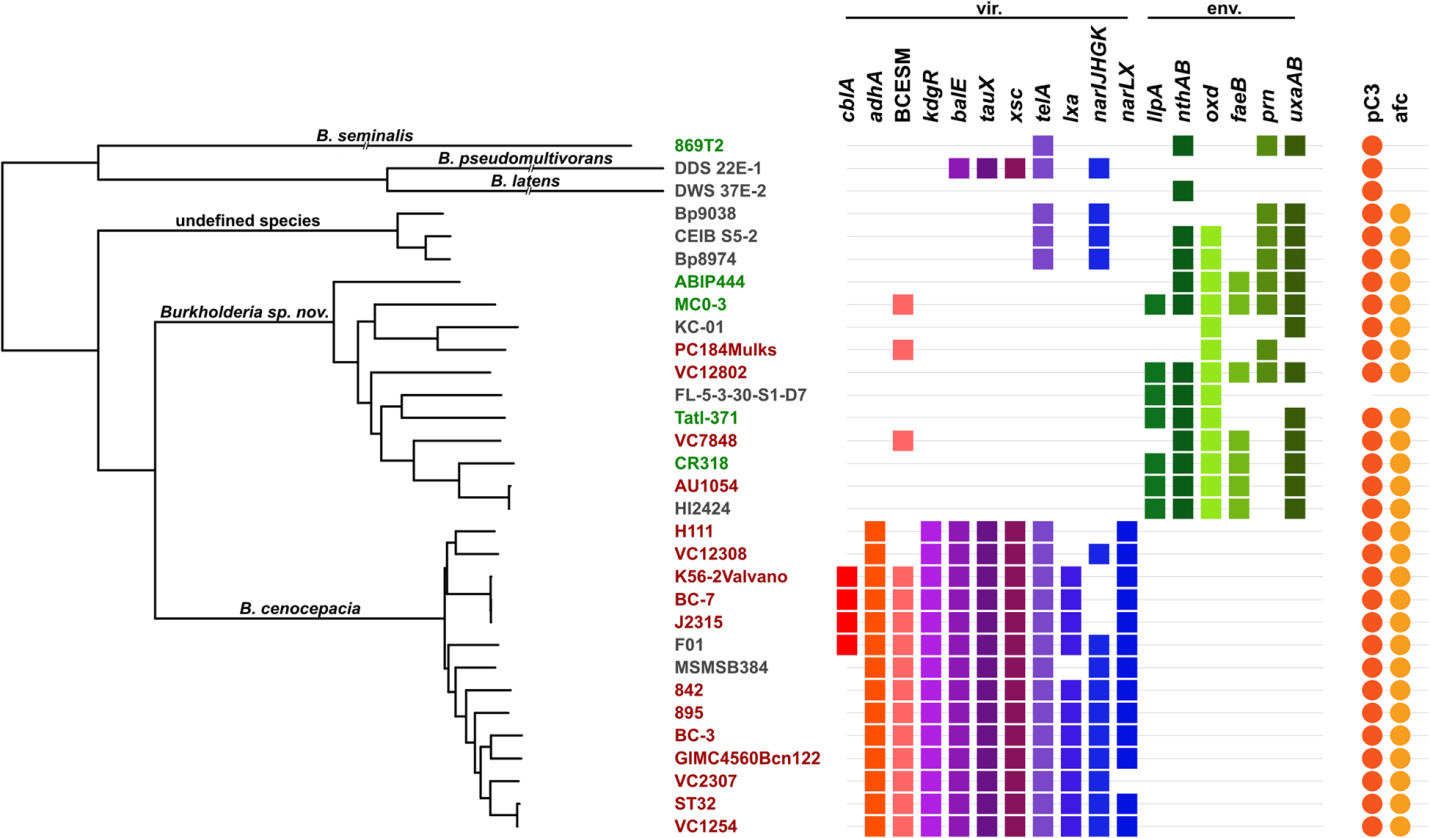
Phylogeny and distribution of host-adaptation genes for 31 *B. cenocepacia* strains. The evolutionary distances were computed using the Maximum Composite Likelihood method. A total of 1057 conserved core-genes, totaling 1,039,265 positions were used in the final dataset. Branch label colors are indicative of the isolation source of the respective strains. These can either be clinic (red), rhizospheric (green) or environmental (grey). The colored shapes indicate the presence of genetic elements in the genomes of the corresponding strains. Squares correspond to genes that were found to be preferably enriched in clinical (vir.) or environmental (env.) species. From left to right: cable pilus (*cblA*), 22 kDa adhesion (*adhA*), *Burkholderia cenocepacia* epidemic strain marker (BCESM), transcriptional regulator *kdgR*, bile acid 7-alpha dehydratase (*baiE*), taurine dehydrogenase (*tauX*), sulfoacetaldehyde acetyltransferase (xsc), tellurite resistance cluster (*telA*), low oxygen activated locus (*lxa*), respiratory nitrate reductase cluster (*narIJHGK*), nitrate sensor and regulation cluster (*narLX*), lectin like bacteriocin 88 (*llpA*), nitrile hydratase cluster (*nthAB*), phenylacetaldoxime dehydratase (*oxd*), feruloyl-esterase (*faeB*), pyrrolnitrin biosynthesis cluster (*prn*), galacturonate metabolism genes (*uxaAB*). Circles indicate the presence of the pC3 megaplasmid and the *afc* cluster. This figure was generated using iTOL [48]

The genomes of 31 *B. cenocepacia* strains were compared and their core-genome extracted (Additional file 4: Table S1; refer to Methods section for details on strain selection). The resulting 1057 conserved genes were aligned and studied in a phylogenetic analysis using the Maximum Likelihood method (Fig. 1).

This result was validated through a Bayesian prediction using the BEAST software (Additional file 2: Figure S2). Both approaches yielded comparable reconstructions. An additional tree resulting from a Neighbor Joining analysis with 1000 bootstrap repetitions is also available (Additional file 3: Figure S3). Among the 31 strains labelled as *B. cenocepacia*, three (869 T2, DDS 22E-1 and DWS 37E-2) fall outside the main clade. The 28 other strains fall within three main clades. One clade gathers the strains belonging to the *recA*-IIIA lineage, including the ET 12 lineage (J2315^T^, BC-7, K56) plus 11 other strains, among which only one was isolated from the environment (F01, from a soil in Burkina Faso). The sister clade of this latter group is composed of 11 strains which belong to the *recA*-IIIB lineage. Seven originate from a plant environment and four from a hospital environment.

The closest outgroup of these two clades contains three strains (Bp8974, Bp9038 and CEIB S5–2) isolated from soil in Mexico and Puerto Rico. These three clades are all extremely well supported by bootstrap values (Additional file 3: Figure S3).

### Whole-genome comparisons

**Fig. 2:**
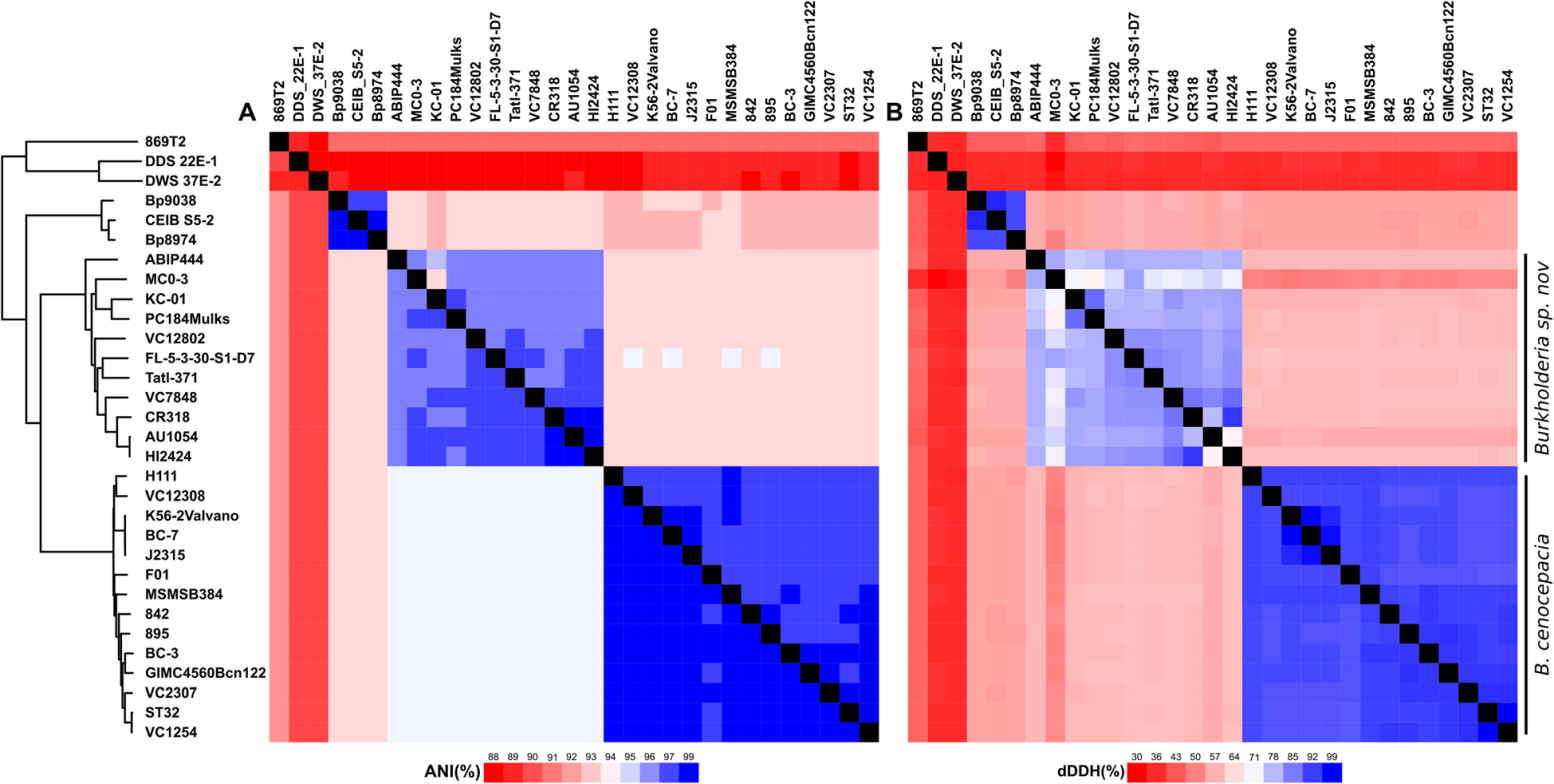
Whole-genome comparisons of 31 *B. cenocepacia* strains. The calculations were performed using the Python module PYANI [49]. Two major identity clusters are formed. The bottom cluster consists of *B. cenocepacia* strains and the second cluster consists of *Burkholderia* sp. nov. strains. One minor identity cluster is formed by the three outlier strains (Bp9038, CEIB_S5–2, Bp8974) and the last three strains are neither genetically related to *B. cenocepacia* nor to each other. A double entry heatmap was used to depict the ANI results with ANIm as left entry and ANIb as right entry (**a**). the dDDH results are depicted on a single heatmap (**b**). The species demarcation threshold is at ≥95% identity on ≥70% aligned genomic sequence for ANI and at ≥70% identity for dDDH. The exact values and sequence cover ratios are available in Additional file 5: Table S2

Based on the ANI analyses and considering the 95% threshold for species delimitation, most input strains cluster in three main species identity groups (Fig. 2a, Additional file 5: Table S2). This distribution is identical to the three clades detected in the previous phylogenetic analyses. The first group includes mainly clinical strains with the exception of strain F01. Consistently, this cluster contains the highly transmissible strains belonging to the ET 12 lineage and can thus be considered as the *B. cenocepacia* *sensu stricto (s.s.)* species. Eleven strains belong to the sister clade of *B. cenocepacia s.s.* and their average nucleotide identity to this latter ranges from 92 to 94% (Additional file 5: Table S2). No closer ANI was found with any of the phylogenetically closest *Burkholderia* species (data not shown). Similarly, the three strains of the third clade do not display any ANI ≥ 95% with *B. cenocepacia*. Their closest *Burkholderia* relative is *B. cenocepacia* strain FL-5-3-30-S1-D7 with 94% ANI value.

Finally, three strains that were originally described as *B. cenocepacia* show closer identity with other species (Additional file 5: Table S2). Strain 869 T2 should be affiliated to the *Burkholderia seminalis* taxon (98.99% identity with 88.85% cover). Strain DDS 22E-1 shares high ANI scores with *Burkholderia pseudomultivorans* (97.57% identity with 80.85% cover) while strain DWS 37E-2 is related to *Burkholderia latens* with 99.01% homology and 89.92% cover.

Two genome alignment methods were used for the ANI analyses, one based on BLAST+ (ANIb) and the other on MUMmer (ANIm). ANIb resulted in a robust species delimitation between *B. cenocepacia* and *Burkholderia* sp. nov. as the values between those clusters are below the 95% threshold. ANIm improved the proximity among species within the clusters. The minimal identity value between *B. cenocepacia* and *Burkholderia sp nov.* strains respectively increased from 94.86 to 97.57% and 97.97 to 98.92%. However, the maximal identity values between the clusters increased as well going from 94.76 to 95.32% and thus passing, although marginally, the threshold value for species delimitation.

The species delimitation was validated through a digital DNA-DNA hybridization (dDDH) analysis (Fig. 2b, Additional file 5: Table S2). For a pairwise comparison between two genomes, a dDDH value ≤70% indicates that the tested organisms indeed belong to different species. Considering this threshold, the species delimitation between *B. cenocepacia* and *Burkholderia* sp. nov. is very well supported with values ranging from 49.9 to 61% .

### General genomic features of *Burkholderia* sp. nov.

**Fig. 3:**
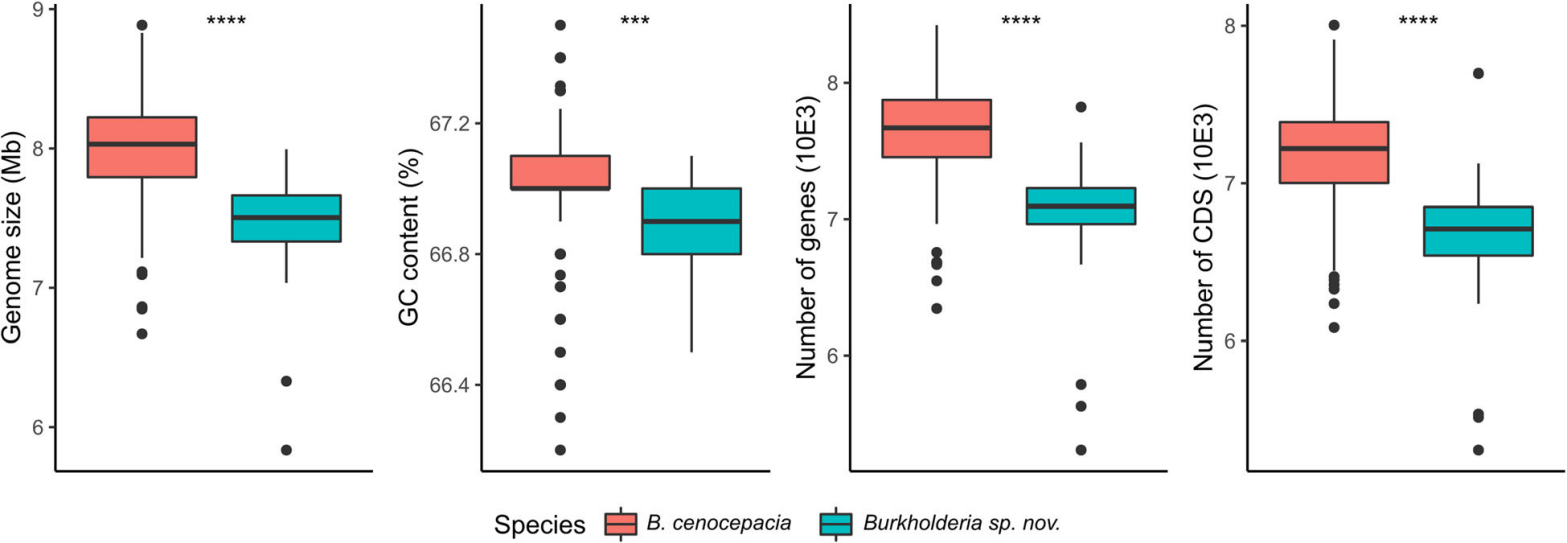
Variations in genomic organization between *B. cenocepacia* and *Burkholderia* sp. nov.. The data of 304 genomes presented in Additional file 6: Table S3 was used to represent the differences in genomic organization between *B. cenocepacia* and *Burkholderia* sp. nov. strains. Significant levels in variations were determined using Student’s t-test (*p* < 2.10^− 4^, *p* < 2.10^− 5^ for *** and **** respectively)

In the following parts, we will refer to the clade harboring in majority environmental strains as *Burkholderia* sp. nov., while isolates that fall together in the same clade as the ET 12 lineage will keep the name *B. cenocepacia s.s.*. Occasionally, the group formed by those two main clades will be referred to as *B. cenocepacia* *sensu* *lato (s.l.)*. The third clade englobes strains with high similarity originating from only two sampling sites and needs to be completed with other isolates from other sites to be confirmed as a new species. As the quality of genome sequences is heterogeneous for the strains used in this study, no comparison of the global genomic architecture was carried out. We screened the strains for the presence of the pC3 megaplasmid containing the virulence associated *afc* cluster [50, 51]. Although we cannot confirm its megaplasmid structure from the draft genomes, large genetic portions of the pC3 were detected in all strains but FL-5-3-30-S1-D7. Strains 869 T2, DDS 22E-1 and DWS 37E-2 harbor a pC3 lacking the *afc* cluster (Fig. 1). On average, *Burkholderia* sp. nov. strains have a slightly, yet significantly (Student’s t-test, *p* < 5.10^− 5^), smaller genome than their closest related species, with a median value of 7.51 Mb as compared to 8.03 Mb for *B. cenocepacia* (Fig. 3). Accordingly, the putative new species has an average of 509 coding sequences less than *B. cenocepacia* with a mean of 6711 and 7220 CDS respectively*.* The GC % content of both species is comparable with approximately 67% (Fig. 3, Additional file 6: Table S3). Nevertheless, both species share a relatively large genome in regards to the genus *Burkholderia* which averages at 7.2 Mb.

### Analysis of core-genome features involved in host-adaptation

We analyzed the core-genome of *B. cenocepacia s.s*. and looked for genes that are strictly absent from the core-genome of *Burkholderia* sp. nov. and reciprocally. This list was further curated from genes with convergent functions when those were successfully annotated. The core-genome of *Burkholderia* sp. nov. comprises 150 genes that are missing from *B. cenocepacia* whereas the latter harbors 244 genes which *Burkholderia* sp. nov. strains do not harbor. These genes sets were further curated from uncharacterized genes which yields 67 core-genes for *Burkholderia* sp. nov and 37 core-genes for *B. cenocepacia* (Additional file 7: Table S4). For both groups, we found several antimicrobial-compound coding genes as well as metabolical genes contributing to environmental competitiveness or improved survival inside their respective hosts. Still, many of the conserved genes play an unknown role. Below, we further elaborate on conserved genes that are susceptible to play a role in specific ecological adaptation. The conservation of these genes of interest across the different taxa and 303 genomes of *B. cenocepacia s.l.* is given in Additional file 8: Table S5.

### Distribution of virulence-associated genes

Based on the literature [50–54], all 31 strains were screened for the presence of several genes previously demonstrated to be involved in virulence (Fig. 1, Table 2). Two well described virulence genes have a striking unbalanced repartition between *B. cenocepacia* and *Burkholderia* sp. nov.: The cable pilus coding gene, *cblA*, is only present in the ET 12 lineage strains and strain F01, and its associated 22 kDa adhesin coding gene, *adhA*, is ubiquitously found among *B. cenocepacia* strains but strictly absent from *Burkholderia* sp. nov. We further focused on candidate genes that are potentially involved in human-host adaptation and specific to *B. cenocepacia* (Table 2). We found a putative bile-acid dehydratase (*BCAM1585–86*), a taurine dehydrogenase (*BCAM1182–83*) and a cluster potentially involved in fatty acid degradation (*BCAM1620–*48).

**Table 2:** List of human virulence-facilitating genes

Gene	Product	Function	Reference
*cblA*	cable pilus	Promotes adhesion to host epithelial cells	[55]
*adhA*	22 kDa-adhesin		[56]
*esmR*	BCESM	Burkholderia cenocepacia epidemic strain marker region	[53, 57]
*amiI*			
*cciI*			
*cciR*			
*opcI*			
*kdgR*	transcriptional regulator of metabolic genes	Can improve virulence	[58–60]
*baiE*	bile acid 7-alpha dehydratase	Putatively involved in a steroid degradation pathway Allows viability within host macrophage	[61–65]
*tauX*	taurine dehydrogenase		
*xsc*	sulfoacetaldehyde acetyltransferase		
*telA*	tellurite resistance protein	tellurite resistance	[66, 67]
*terCEF*	integral membrane protein		
*narIJHG*	nitrate reductase gamma subunit	anaerobic metabolism through nitrate reduction	[68]
*narL*	DNA-binding response regulator		
*narX*	Nitrate/nitrite sensor protein		
*lxa*	low oxygen activated locus	maintains cell viability after oxygen depletion	[69]

We also searched for genes putatively involved in defense against the host immune system but also in host specific resilience and virulence. In those categories *B. cenocepacia* specifically possesses resistance genes towards tellurite (*BCAL2268–*71) as well as adaptation genes towards anaerobic metabolism. Considering the different pathways and components potentially allowing anaerobic survival of bacteria (Fig. 1, Table 2), only *B. cenocepacia s.s.* and the third cluster harbor the required operon for respiratory nitrate reduction (*narIJHGK*). Within *B. cenocepacia*, this operon is absent from the ET 12 lineage and strain H111 (Fig. 1, Table 2). Still, all *B. cenocepacia* *s.s.* strains possess the genes coding for the nitrate/nitrite sensor (*narX*) and the associated regulator (*narL*). However, the gene clusters necessary for subsequent respiratory reduction of nitrite, nitric-oxide and nitrous-oxide are missing in every *B. cenocepacia* *s.l.* strain. The *lxa* genomic island spans over 50 genes and is involved in cell viability after oxygen depletion [69]. This cluster was detected in most *B. cenocepacia* strains (*BCAM0275a-323*) as well as in those of the third cluster, but was completely lacking or missing vast genetic portions (at least 37% of the total cluster length) in *Burkholderia* sp. nov. strains.

### Distribution of plant-adaptation and environmental-resilience genes

We investigated the presence of five genes or gene clusters which are, according to previous studies, involved in improving the fitness of plant associated bacteria [70, 71]. Two genes involved in defense strategies were detected in *Burkholderia* sp. *nov*., the lectin-like bacteriocin LlpA-88 (*Bcen_1091*) and the antifungal antibiotic pyrrolnitrin (*Bcenmc03_6983–86*).

Regarding metabolic features, *Burkholderia* sp. nov. strains were found to possess several enzymes such as a nitrile hydratase (*Bcen_4082–85*), a phenylacetaldoxime dehydratase (*Bcen_4078–*81), a feruloyl-esterase (*Bcen_1301*) and a galacturonate metabolism operon (*Bcen_6467–68*) allowing these bacteria to catabolize plant derivatives. It is important to point out that numerous additional plant-adaptive genes are present in the genomes of *Burkholderia* sp. nov. strains. However, these genes are not addressed here as they are shared with *B. cenocepacia* strains.

### Evolutionary history of *B. cenocepacia*

The clade, formed by the third identity cluster, possesses several plant-adaptive traits which are part of the *Burkholderia* sp. nov. specific core-genome (i. e. nitrile hydratase, phenylacetaldoxime dehydratase, pyrrolnitrin synthase) (Fig. 1, Table 3). Conversely, these isolates do not possess any of the investigated genes suggested to confer a direct advantage to *B. cenocepacia* during human infection (i.e. BCESM, *cblA*, *adhA*) (Fig. 1, Table 2). This observation can be extended to the outgroup species *B. seminalis, B. latens* and *B. pseudomultivorans*.

**Table 3:** List of genes improving plant interaction and environmental fitness

Gene	Product	Function	Reference
*nthAB*	nitrile hydratase	Metabolism of plant derivatives and/or IAA synthesis pathway	[72, 73]
*oxd*	phenylacetaldoxime dehydratase		[73, 74]
*llpA*	lectin-like bacteriocin	Antibiotic	[26]
*faeB*	feruloyl-esterase	Metabolism of plant derivatives	[75, 76]
*prnA-D*	pyrrolnitrin	Antibiotic	[77]
*uxaAB*	altronate dehydratase/oxydoreductase	Galacturonate metabolism	[78]

The phylogenetic reconstructions also support a different pattern of molecular evolution between the two main clades. The reconstruction shows a longer branch leading to *B. cenocepacia* followed by short inner branches. *Burkholderia* sp*. nov* displays an opposite pattern, with a shorter basal branch and longer inner branches (Fig. 1, Additional file 2: Figure S2 and Additional file 3: Figure S3).

## Discussion

### *B. cenocepacia* strains have a polyphyletic organization

Regardless of the method used for their genomic comparisons (phylogenetic and ANI analyses), the results yielded a disunited perspective of the *B. cenocepacia* species (Figs. 1 & 2). The ANIb and dDDH analyzes yielded strong separations of the different clades based on the conventional threshold values for species delimitation. While the ANIm analysis strengthened the proximity within the clusters, it did not provide as clear differences between *B. cenocepacia* and *Burkholderia* sp. nov. as the two previous approaches. However, given the results from the remaining whole-genome comparison methods and the MLSA approach, we are confident that our results showed that the *B. cenocepacia* taxon should be split in two or possibly three distinct species (not considering three strains for which we proved a clear false taxonomic attribution).

Here, we propose to keep the *B. cenocepacia* name for all strains clustering with the epidemic ET 12 lineage, representing *B. cenocepacia* in its most studied state, as a potential human opportunistic pathogen. We further propose to reclassify its sister clade, *Burkholderia* sp. nov., as a new species (see below for a suggested name description). The third cluster, sister clade of the two latter species could also represent a novel *Burkholderia* species and deserve to be investigated independently. Still, more sampling is needed since the species are very similar to each other and were isolated from only two different geographic areas (Additional file 1: Figure S1).

Unexpectedly, four clinical isolates, namely AU1054, PC184Mulks, VC12802 and VC7848, fall in the *Burkholderia* sp. nov. clade. As a first hypothesis, these strains could have survived in a clinical environment solely as commensal bacteria, causing no harm to their host. The previous isolation of *Burkholderia* strains from CF patients or patients suffering from another infectious pathology, without being the apparent causative agent supports this hypothesis [79–81]. Although strain AU1054 was shown to cause high mortality rates in diverse plant and animal models [50, 82], this is not sufficient to assess its pathogenesis against humans. Indeed, strain H111, which belongs to the *B. cenocepacia* *s.s.* clade, presents the same characteristics as AU1054 on several pathogenesis models [83] and yet does not cause any symptoms in humans. When detected in patients, the bacterial population of H111 decreases over time, unable to maintain itself in a CF context [84]. At this point, we cannot exclude the alternative hypothesis that these clinical strains are in fact human opportunists. However, as discussed in the next section, these isolates lack many key virulence traits that are present in *B. cenocepacia s.s.*. Still, more virulence and pathogeny tests are needed on strains of this clade to fully rule out their potential human virulence.

### *B. cenocepacia* possess specific key virulence traits compare to *Burkholderia* sp. nov

Over the past years, many genetic markers have been investigated for their involvement in *B. cenocepacia* pathogenesis. When mapping some of these virulence factors, the most striking pattern in the distribution between the two clades concerns the *adhA* and *cblA* genes. Both cable pilus and the associated 22-kDa adhesin have been shown to be involved and decisive for host cell binding [55, 56]. Their distributions are congruent with previous studies that found *adhA* to be mandatory and sufficient for cell binding but *cblA* required for optimal binding [54]. Our data supports that the presence of *adhA* is essential for the opportunistic potential of *B. cenocepacia* strains.

Additionally, a genomic island termed *B. cenocepacia* epidemic strain marker (BCESM) was frequently found in CF isolates [53]. However, it was demonstrated that the BCESM is not an absolute marker for the ability of *B. cenocepacia* to cause CF infection [57]. In our study, the distribution of this cluster is less clear, as it was detected at a lower frequency in the *Burkholderia* sp. nov. strains compare to *B. cenocepacia* (58 and 83% respectively, Additional file 8: Table S5). This difference might just be random, but we believe that it reflects a specialization trend of the BCESM cluster for pathogenicity.

As a counter example, the pC3 megaplasmid was detected in every *B. cenocepacia* strain and all but one *Burkholderia* sp. nov. strains. This replicon was shown to play an important role in the pathogenesis of various BCC strains, including *B. cenocepacia*, in diverse infection models [50]. Located on this pC3, the *afc* cluster and the adjacent transcriptional regulator *shvR* were recently demonstrated to be required for acute infection of *B. cenocepacia* in the zebrafish model [51]. However, the virulence levels of *B. cenocepacia* strains are strongly depending on the host model. To date, strains of *B. cenocepacia s.s.* and *Burkholderia* sp. nov. have been tested on nematodes, wax moth larvae and zebrafish and the strains respective virulence sometimes varied drastically depending on the tested host [51, 85, 86]. Tests that aim to study the human infection route through the lungs have only been performed with ET-12 representatives and we support that clinical research would greatly benefit if a wider diversity of strains including *Burkholderia* sp. nov. representatives were tested in future studies*.*

The following genes have not yet been described in *B. cenocepacia* but their homology to virulence factors of other pathogens might indicate a similar role in our species of interest.

While analyzing the specific core genome of each clade, we detected that *Burkholderia* sp. nov lacks two *B. cenocepacia* specific enzymes, a bile-acid dehydratase and a taurine dehydrogenase. Bile acids can be conjugated to taurine before they are secreted inside the digestive track. Furthermore, bile acids are cholesterol-derivatives formed by conversion in the liver [87] and it has been previously demonstrated that several human pathogens could thrive on cholesterol compounds as a carbon source [61, 62]. *Mycobacterium tuberculosis* is able to maintain itself within host macrophages through this same steroid degradation pathway [63]. Thus, in addition to an improved ability to bind epithelial cells, *B. cenocepacia* also displays key features that could promote its survival inside host cells as compared to *Burkholderia* sp. nov..

A resistance gene for tellurite is also present in the core-genome specific to *B. cenocepacia*. This antimicrobial compound has strong oxidizing abilities but is also believed to be a substitute for sulfur in various cellular functions with drastic outcomes for cell metabolism. Tellurite resistance is widespread in pathogenic bacteria and was taken advantage off for generic screening of pathogens using tellurite in selective media [88]. More recently, the human pathogen *Yersinia pestis* was shown to express several tellurite resistance genes during macrophage infection and subsequent studies led to speculate that these genes are part of a bacterial adaptive strategy to macrophage associated stress [66, 67].

Finally, in a CF endobronchial context, important amounts of mucus are secreted, leading to partial or total anaerobiosis [89]. Several bacterial species, such as *P. aeruginosa* or the *B. cenocepacia* related *Burkholderia pseudomallei*, can maintain themselves in micro-oxic conditions through denitrification [90, 91]. The complete respiratory nitrate reduction process works sequentially and involves genes responsible for the reduction of nitrate, nitrite, nitric-oxide and nitrous-oxide [92]. *B. cenocepacia* is usually considered to be an obligate aerobic non-fermenting bacterium, and doesn’t possess all the required genes to perform the complete nitrate reduction reaction. However, most *B. cenocepacia s.s.* strains have the genetic material to use nitrate as electron acceptor and transfer the resulting nitrite from the cytoplasm to the periplasm. This chain reaction allows the transfer of 2 H^+^ from the cytoplasm to the periplasm. These protons can be thereafter used for ATP synthesis. An additional specificity of *B. cenocepacia* over *Burkholderia* sp. nov. is the presence of the *lxa* locus. This 50 gene cluster is crucial for the bacterium’s survival in low oxygen conditions [69]. It should be noted that the lack of both respiratory nitrate reduction and the *lxa* cluster in H111 might explain why this strain cannot maintain itself in CF patients [84]. Overall, *Burkholderia* sp. nov. lacks several adaptation genes for survival in anaerobic environments.

Taken together, the distribution of these pathogenic or adaptive genes showed a strong tendency in favor of an adaptation of *B. cenocepacia* strains for human pathogenicity compared to *Burkholderia* sp. nov. strains.

### *B. cenocepacia* lacks environmental traits present in *Burkholderia* sp. nov.

*Burkholderia* sp. nov. encompasses mostly strains that have been retrieved from diverse environmental samples, including healthy plant roots. These bacteria are under the selection of a fluctuant environment and heavy competition from other microorganisms that thrive in these niches. Following this view, several strains of *Burkholderia* sp. nov. possess the gene cluster coding for the lectin-like bacteriocin LlpA88 and for the antifungal metabolite pyrrolnitrin. Both these metabolites have a very broad-spectrum antibiotic activity [26, 93].

Adaptation to these constraints also requires the ability to exploit various nutriments, including root exudates. When mining in the core-genome of *Burkholderia* sp. *nov*., but also the core genome of the third identity clade, we detected two genes supporting a clear adaptation to the plant environment. The first is a nitrile hydratase allowing these bacteria to use nitrile as carbon and nitrogen source. Several natural sources for nitrile are known and many are plant-based [94]. In the same genomic cluster, a gene coding for a phenylacetaldoxime dehydratase was also found. Aldoximes are volatile plant-derived metabolites which can be used as defense mechanisms against insect herbivores and pathogens [74]. Moreover, phenylacetaldoxime dehydratase is very efficient at catalyzing the production of phenylacetonitril which could be further used by the previously described nitrile hydratase [72]. Alternatively, both enzymes can be involved in indole-3-acetic acid (IAA) production, which is one of the traits that is commonly used to screen for endophytic and rhizosphere competent bacteria [73, 95, 96]. One additional advantage of *Burkholderia* sp. nov. on *B. cenocepacia* in the effective use of rhizodeposits as carbon source may come from a feruloyl-esterase. This enzyme liberates polysaccharides, such as xylan, from its bond with ferulic acid as it is commonly found in plant cell walls [75]. One other major component of plant cell walls is pectin, a polymer of galacturonic acid. *Burkholderia* sp. nov. possess the import and catabolism genes to process D-galacturonate and use it as a carbon source [78].

Genes associated with adaptation to the soil environments can be highly multifarious and are more elusive than genes conferring adaptation to a single niche such as the human body. A more profound analysis is required to determine how efficient and how generalized environmental adaptation is for *Burkholderia* sp. nov. strains.

**Fig. 4:**
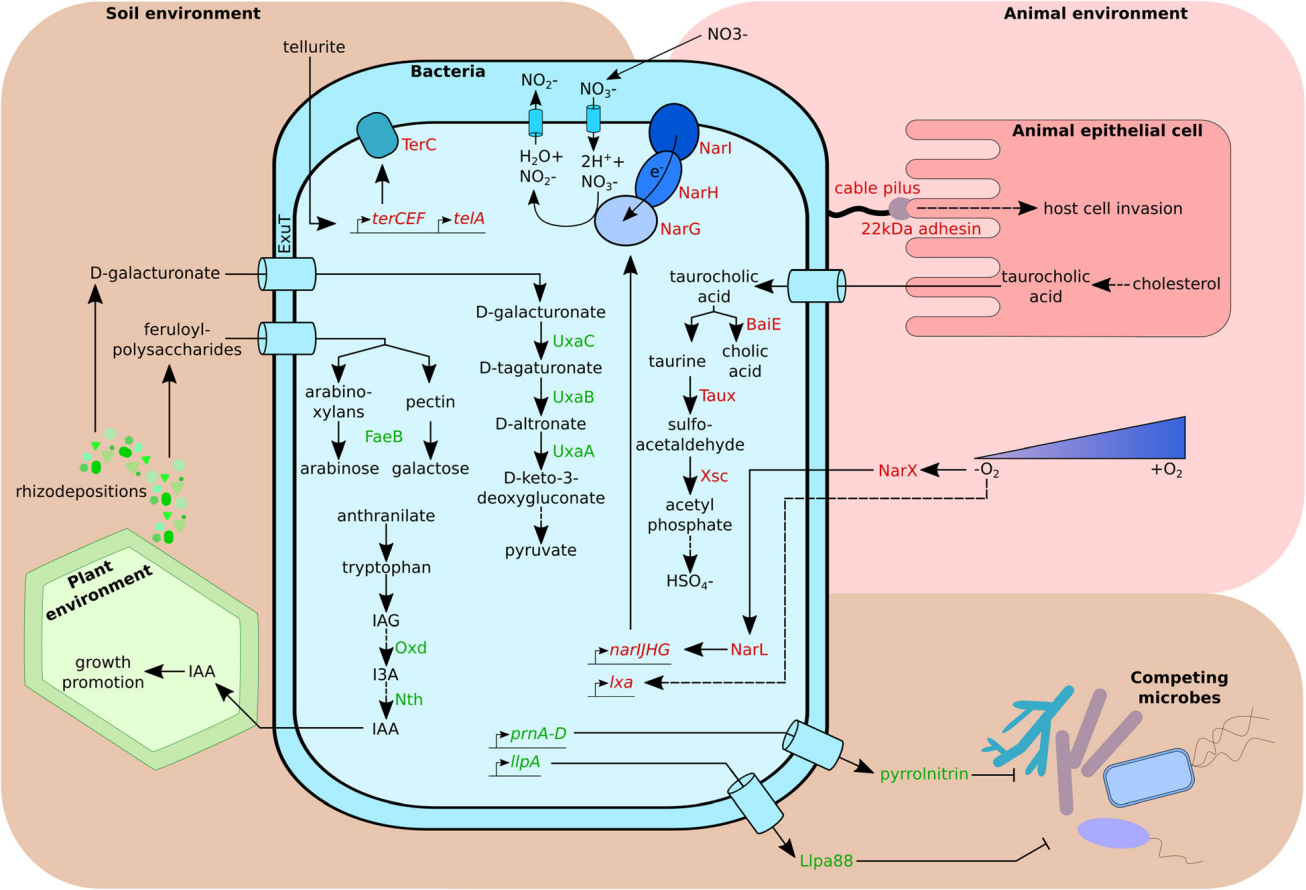
Summary diagram of differential adaptation of *B. cenocepacia* and *Burkholderia* sp. nov. to different environments. Strains of *B. cenocepacia* and *Burkholderia* sp. nov. have been isolated from soils (brown), where they compete with other microbes, plants (green) and animals (red). *Burkholderia* sp. nov. was repeatedly isolated from soil environment (but also plants, water and aerosols) and possesses several genes improving its fitness in those contexts (green factors). While *B. cenocepacia* can also thrive in soils, it is often found as an opportunistic pathogen of humans and bears several genes improving its virulence (red factors). *Burkholderia* sp. nov. can use different plant derivatives (galacturonic acid, xylans, pectin) as carbon sources. It is also proposedly able to synthetize the plant hormone auxin (IAA) through a pathway involving Oxd to convert indole-3-acetaldoxime (IAG) to indole-3-acetonitrile (I3A) which is processed to IAA though the action of Nth. It is also able to produce antibiotics with activity against bacteria (Llpa88) and fungi (pyrrolnitrin). *B. cenocepacia* strains possess a 22 kDa adhesin which improves its binding to target cells and their invasion. Proposedly, they can also metabolize bile acids, derivatives of cholesterol. In anoxic conditions, *B. cenocepacia* can survive using its low oxygen activated locus (*lxa*) and the respiratory nitrate reduction pathway (*narIJHG*). It also possesses the resistance genes against tellurite, for which the exact functions remain elusive. Source: authors’ design

Figure 4 summarizes the discussed genes and functions conferring *Burkholderia* sp. nov and *B. cenocepacia* specific ascendancy in soil environments and human infections respectively.

### The opportunistic pathogens might have evolved from plant-adapted bacteria

One approach to determine the ancestral state of the two clades of *B. cenocepacia* and *Burkholderia* sp. *nov*. is to focus on the outgroup life style. The conservation of environment and plant adaptive traits and the absence of key virulence genes in strains of the third identity cluster and more distant outgroup species, indicate that *B. cenocepacia* might have evolved from a plant-associated life style towards a human opportunist state.

The pattern observed using the phylogenetic reconstruction, reflects a significant evolutionary trend. It shows a lower within-species diversity in *B. cenocepacia* which may have undergone a rapid evolution, by either positive selection and/or population bottleneck, before diversification. Following this view, the core-genome of *B. cenocepacia* strains is higher than that of *Burkholderia* sp. nov. (69.9 and 49.7% of the average amount of genes across strains respectively), confirming that *B. cenocepacia* is less diversified than *Burkholderia* sp. nov.. This might again be a sign for higher selective pressure applied by its environment on *B. cenocepacia* during a pathogenic lifestyle, and/or a more recent evolution, leaving less time for genetic divergence. This would implicate that *B. cenocepacia* has evolved the human-pathogen trait after its ancestors had adapted for interactions with plants.

### Proposition of *Burkholderia servocepacia* sp. nov.

Proposition of name *Burkholderia servocepacia* sp. nov. (*servocepacia*: ser.vo.ce.pa’cia. M.L. v. *servare*, protect / guard; specific epithet, *cepacia*; N.L. n. *servocepacia*, protecting cepacia). The prefix “ceno” from the Greek “kainos” was initially chosen to mean “new” since *B. cenocepacia* was a derivative of the *B. cepacia* species. Interestingly, the Latin meaning of the same prefix is “to dine”. Given that *cepa* is Latin for onion, inspired by the isolation source of the first *Burkholderia* species, the combination *ceno-cepacia* can be understood as “feasting on onions”. This interpretation also has the benefit of harmonizing the etymological origins of both components. Additionally, this predatorily name is in accordance with the serious harm the bacteria can cause in humans. As a sequel, we propose the name *Burkholderia servocepacia* sp. nov. coined from the same epithet and using as a prefix the Latin “servo” meaning “to protect/watch over” in regard of the biocontrol potential of this species.

We propose that *B. servocepacia* Tatl-371 be used as type strain. Its phenotype has been extensively characterized and it was deposited in the BCCM/LMG database under accession number LMG30279 [26]. As a type strain should be representative of its species, it is relevant that Tatl-371 possesses a central position in the phylogeny of its clade (Fig. 1).

## Conclusion

The recurrent isolation of *B. cenocepacia* from plant environments was the premise for the investigation of 31 *B. cenocepacia* strains using comparative genomics.

Despite the nature of plants as environmental reservoir for diverse human pathogens, our study suggests that multiple *B. cenocepacia* affiliated strains are instead specialized for an environmental lifestyle and interactions with plants, and potentially unfit to colonize and cause harm to humans. We demonstrate that those strains are phylogenetically distinct from *B. cenocepacia s.s.* and should be affiliated to a new species. We also suggest that *B. cenocepacia* has acquired the human opportunist trait from a plant adapted basis. *B. cenocepacia* is not a united group at the genomic level. This disunity has high chances to impact the metabolism and the virulence of these bacteria, especially considering the specific or over-represented genes we detected in each clade. We hope that these insights will open promising leads in research on virulence promoting factors of *B. cenocepacia*. It would be most noteworthy if future studies were to consider the large diversity of the cenocepacia group and especially in lung infection models. This study is one of many example that underlines the benefits of interdisciplinary research and in the present case, between environmental microbiology and health sciences. Enhanced communication and collaboration between disciplines is a worthy pursuit which can positively impact the global knowledge.

## Methods

### Bacterial strains & genome sequencing

For the 303 *B. cenocepacia* genomes, publicly available at the time of the study, the *recA* sequences were retrieved (except for VC5279, no *recA* sequence in genomic data) and used to build a phylogenetic prediction using the Maximum Composite Likelihood method (1000 bootstrap repetitions). The resulting tree is available as Additional file 1: Figure S1 and in interactive form online: https://itol.embl.de/tree/912033414356601565192630#. We chose 31 different strains, representative of the diversity in source of isolation. We prioritized fully annotated genomes and included draft genomes when those increased the global diversity. All genomes from plant- or rhizosphere-isolated strains, available at the time of the study, were included in the analyses. All genomes from environment isolated strains outside the *recA*-IIIA clade were also included, plus 2 of the 5 genomes that fall in the *recA*-IIIA clade.

Three hundred one of the bacterial genomes studied here are publicly available on the NCBI database. One strain, CEIB S4–3, is publicly available on the JGI website. The respective strains are all registered as belonging to the *B. cenocepacia* species and are listed in Additional file 6: Table S3 with additional genomic data.

Strain ABIP444, was isolated during a survey of rice roots endophytes in Cameroon (E. Ngonkeu, unpublished). A single colony was grown in Luria low salt medium (Merck, Inc., Darmstadt, Germany) for 24 h at 28 °C and its DNA was extracted using a modified JGI protocol for bacterial DNA isolation using CTAB. A genomic library with average insert size of 350 bp was prepared for sequencing using a TruSeq Nano DNA library preparation kit (Illumina, Inc., San Diego, CA, USA). Paired-end sequencing was performed by the MGX platform (CNRS, Montpellier, France) using a HiSeq 2500 (Illumina), generating 12,709,495 raw read pairs. Reads with overlapping sequence were assembled using CLC Genomics Workbench version 7.04 resulting in 292 contigs ranging from 586 bp to 20.2 kbp. The genome sequences can be found at the European Nucleotide Archive with accession number PRJEB31911.

### Core-genome calculation

The general core-genome of the 31 strains labeled as *B. cenocepacia* was retrieved from protein sequences using the Roary pipeline [97]. Protein clusters are initially produced with CD-HIT using iterations with similarity thresholds going from 100% down to 98% with 0.5% decrements. An all-against-all comparison is performed on the resulting set of protein sequences with BLASTP at 95% sequence identity. The genome annotations and GFF3 format files were obtained through the Prokka pipeline [98].

Further analysis of the data generated by core-genome calculation (described below), identified two main clades. Their specific (i.e. strictly absent from the neighbor clade) core-genome (gene conservation across ≥80% of strains) were generated using the Roary pangenome output files.

### Genomic comparisons

Pairwise genome comparison using Average Nucleotide Identity (ANI) based on BLAST+ (ANIb) or MUMmer (ANIm) was performed using the PYANI software [49]. Two strains were considered co-specific when they shared more than 95% nucleotide identity on at least 70% of their whole genome sequence [99]. For the strains that did not fall within a clear ANI cluster, comparisons to the phylogenetically closest *Burkholderia* species were carried out [1].

Digital DNA-DNA hybridization was carried out using the GGDC 2.1 web platform [100]. Briefly, this tool uses BLAST+ to align one genome against another and reciprocally and generate high-scoring segment pairs (HSPs). The intergenomic distance is then calculated by dividing the sum of all identities found in HSPs by overall HSP length. A logistic regression is used for reporting the probability that the distance value is above 70%, the similarity threshold to consider two organism as distinct species. Compared to ANI, dDDH can offer more robust estimations of genomic similarities for incomplete genomes as the distance calculation is independent of genome length.

### Phylogenetic analysis

The phylogenetic analyses were conducted using the MEGA v7.0.26 software [101]. The core-genome output of 1057 genes (1,039,265 positions) was evaluated using a Maximum Likelihood phylogenetic reconstruction approach with a General Time Reversible model (Gamma distributed rates with invariant sites and 5 discrete gamma categories). We validated the resulting tree through an independent Bayesian analysis using BEAST v1.10.2 [102], with the same substitution model, a strict clock model and the tree prior calculated using the Yule model. The MCMC length of chain was set to 1.10^7^ and the burn-in value was set to 1.10^6^ for analysis. The resulting consensus tree was constructed using a maximum clade credibility prediction.

### Host-adaptive gene detection

The specific core genomes of the two main clades, were screened for genes described as involved in host adaptation in the literature [50–54, 70, 71]. The complete sequence of the 875 kbp long virulence plasmid from *B. cenocepacia* J2315 and the 24 genes of the *afc* pathogenic cluster were also searched in all 31 genomes by BLAST [50, 51].

Gene clusters involved in anaerobic metabolism, showing potential adaptation to CF lung environments, were identified through similarity with their *B. pseudomallei* homologues [68]. We also searched for the low-oxygen-activated locus (*lxa*) which was discovered in *B. cenocepacia* [69].

The antiSMASH 4.0 software [103] was used to screen for gene clusters allowing the production of secondary metabolites such as antibiotics and siderophores, involved in environmental resilience and competition with other microorganisms.

The specificity to one taxa was validated for each gene through its alignment against a database of the 303 available *B. cenocepacia s.l.* sequences using BLAST.

At the first occurrence of each genetic element, we provide its gene name in UniProt format. When possible, we use the gene name of the *B. cenocepacia* type strain J2315. When the gene is absent from the type strain, the next best annotated genome is used.

## Supplementary information

**Additional file 1: Figure S1.** *recA* based phylogeny for 302 *B. cenocepacia* strains. The *recA* sequence of *B. pseudomallei* K96243 was used to root the tree. The evolutionary history was inferred using the Neighbor-Joining method. The associated taxa that clustered together in > 95% of replicate trees in the bootstrap test (1000 replicates) are displayed as black dots on the tree branches. The color ranges delineate the two recA lineages: IIIA (orange) and IIIB (blue). The colored strip indicates the isolation source (when known) of the respective strains: clinical (red), plant (green) and environmental (grey). The strains which were included in whole-genome analyses are marked by a black arrowhead. The outermost blue histogram is representative of the genomic completeness for the respective strains according to the NCBI annotation. In increasing bar size order: contig, scaffold, chromosome and complete. An interactive version in full quality is available online (https://itol.embl.de/tree/912033414356601565192630#).

**Additional file 2: Figure S2.** BEAST-generated phylogenetic tree of 31 *B. cenocepacia* strains. A Bayesian analysis using the BEAST v1.10.2 software was used to generate this tree. The input data is the same as for Fig. 1. A General Time Reversible model (GTR; Gamma distributed rates with invariant sites and 5 discrete gamma categories) was used as substitution model. A strict clock model was applied and the tree prior was calculated using the Yule model. Finally, the MCMC length of chain was set to 1.10^7^ and the burn-in value was set to 1.10^6^ for analysis. The resulting consensus tree showing mean branch lengths was constructed using a maximum clade credibility prediction.

**Additional file 3: Figure S3.** Phylogenetic tree of 31 B. cenocepacia strains. The input data is the same as for Fig. 1. The evolutionary history was inferred using the Neighbor-Joining method. The associated taxa that clustered together in > 95% of replicate trees in the bootstrap test (1000 replicates) are displayed as black dots on the tree branches. The tree is drawn to scale. The evolutionary distances were computed using the Maximum Composite Likelihood method and are in the units of the number of base substitutions per site. The analysis involved 31 nucleotide sequences. All positions with less than 95% site coverage were eliminated. That is, fewer than 5% alignment gaps, missing data, and ambiguous bases were allowed at any position. There were a total of 1,118,599 positions in the final dataset.

**Additional file 4: Table S1.** Core-genome of the 31 *B. cenocepacia* strains. The 1065 genes used in the MLSA approach are detailed with their function, average gene length and nucleotide sequence.

**Additional file 5: Table S2.** Detailed whole genome comparison data. Percentage identity and percentage coverage values for the ANIb and ANIm approaches as well as percentage identity for the dDDH approach. Taxonomic classification of strains 869 T2, DS 22E-1 and DWS 37E-2 based on their genomic alignment with *B. seminalis*, *B. pseudomultivorans* and *B. latens* respectively as estimated by ANIb.

**Additional file 6: Table S3.** Additional genomic information on all available *B. cenocepacia* strains. Each strain is detailed with its specific affiliation, genome completeness, genomic size, GC content, genomic architecture, number of genomic scaffolds, gene count and protein count. The 31 strains used in the study are highlighted in yellow.

**Additional file 7: Table S4.** Specific core-genomes of *B. cenocepacia* and *Burkholderia* sp. nov. Each gene is detailed with its function, abundance within the species and average length. Their presence within a genome is marked with an “X”. The genes further described in the study are highlighted in yellow.

**Additional file 8: Table S5.** Distribution across taxa of virulence facilitating and environmental fitness improving genes. For each gene, their product and their function in virulence or environmental fitness are described. The nucleotide sequence of each gene was aligned against a database of 304 *B. cenocepacia* genomes using BLAST. The percentage of occurrence among strains of each major taxon (*B. cenocepacia*, *Burkholderia* sp. nov. and the third undefined taxon) is given. The table also shows if a gene was found in a least one outgroup strain.

## Data Availability

301 *B. cenocepacia* genomes analyzed during the current study are available in the NCBI repository (https://www.ncbi.nlm.nih.gov/genome/genomes/475). Strain CEIB S4–3, not currently annotated as *B. cenocepacia*, is available under RefSeq accession number JSBM00000000.1. The novel sequenced strain ABIP444 is available at the European Nucleotide Archive under study accession number PRJEB31911, http://www.ebi.ac.uk/ena/data/view/PRJEB31911. The datasets used and/or analysed during the current study are available from the corresponding author on reasonable request.

## Abbreviations

ANI: Average Nucleotide Identity
BCC: *Burkholderia cepacia* complex
BCESM: *Burkholderia cepacia* epidemic strain marker
CF: Cystic fibrosis
dDDH: Digital DNA-DNA hybridization
IAA: Indole-3-acetic acid
