# Supplementary figures and images for: Genomic analyses of Burkholderia cenocepacia reveal multiple species with differential host-adaptation to plants and humans
Source: BMC Genomics. 2019 Nov 4;20:803. doi: 10.1186/s12864-019-6186-z (PMC6829993; doi:10.1186/s12864-019-6186-z)

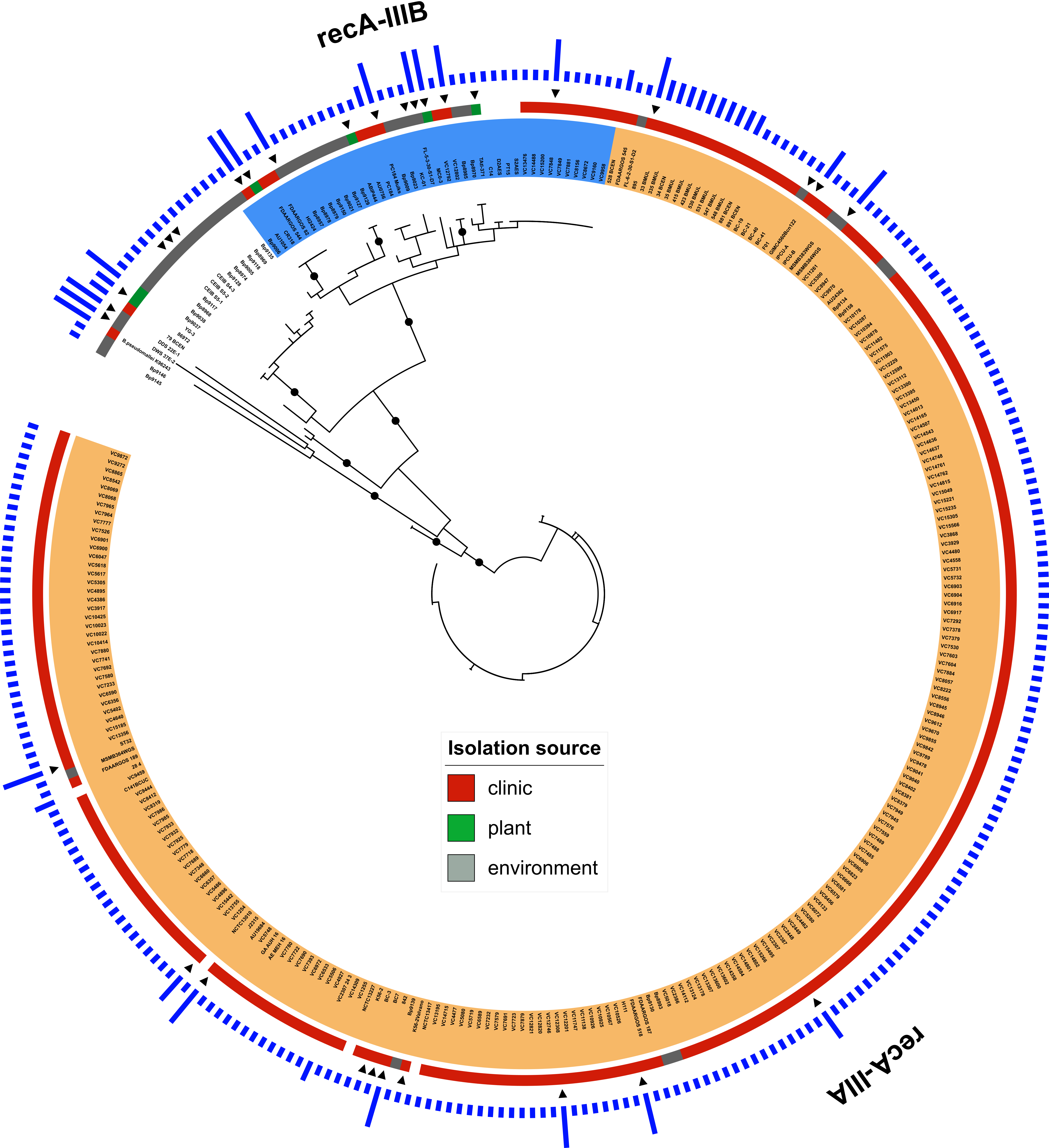

Supplement: Supplementary file 1 — Additional file 1: Figure S1. recA based phylogeny for 302 B. cenocepacia strains. The recA sequence of B. pseudomallei K96243 was used to root the tree. The evolutionary history was inferred using the Neighbor-Joining method. The associated taxa that clustered together in > 95% of replicate trees in the bootstrap test (1000 replicates) are displayed as black dots on the tree branches. The color ranges delineate the two recA lineages: IIIA (orange) and IIIB (blue). The colored strip indicates the isolation source (when known) of the respective strains: clinical (red), plant (green) and environmental (grey). The strains which were included in whole-genome analyses are marked by a black arrowhead. The outermost blue histogram is representative of the genomic completeness for the respective strains according to the NCBI annotation. In increasing bar size order: contig, scaffold, chromosome and complete. An interactive version in full quality is available online (https://itol.embl.de/tree/912033414356601565192630#). [file 12864_2019_6186_MOESM1_ESM.png]

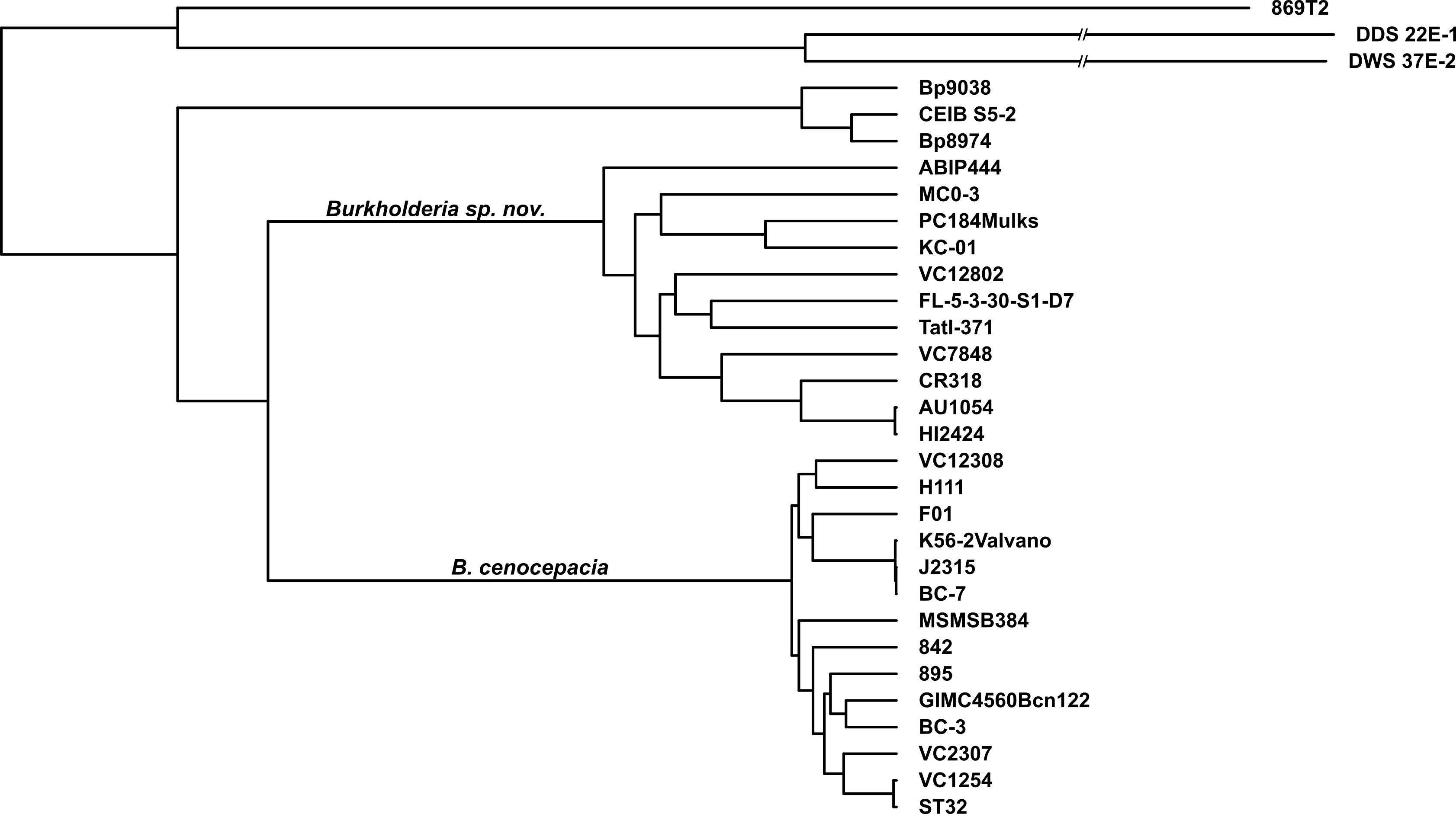

Supplement: Supplementary file 2 — Additional file 2: Figure S2. BEAST-generated phylogenetic tree of 31 B. cenocepacia strains. A Bayesian analysis using the BEAST v1.10.2 software was used to generate this tree. The input data is the same as for Fig. 1. A General Time Reversible model (GTR; Gamma distributed rates with invariant sites and 5 discrete gamma categories) was used as substitution model. A strict clock model was applied and the tree prior was calculated using the Yule model. Finally, the MCMC length of chain was set to 1.107 and the burn-in value was set to 1.106 for analysis. The resulting consensus tree showing mean branch lengths was constructed using a maximum clade credibility prediction. [file 12864_2019_6186_MOESM2_ESM.png]

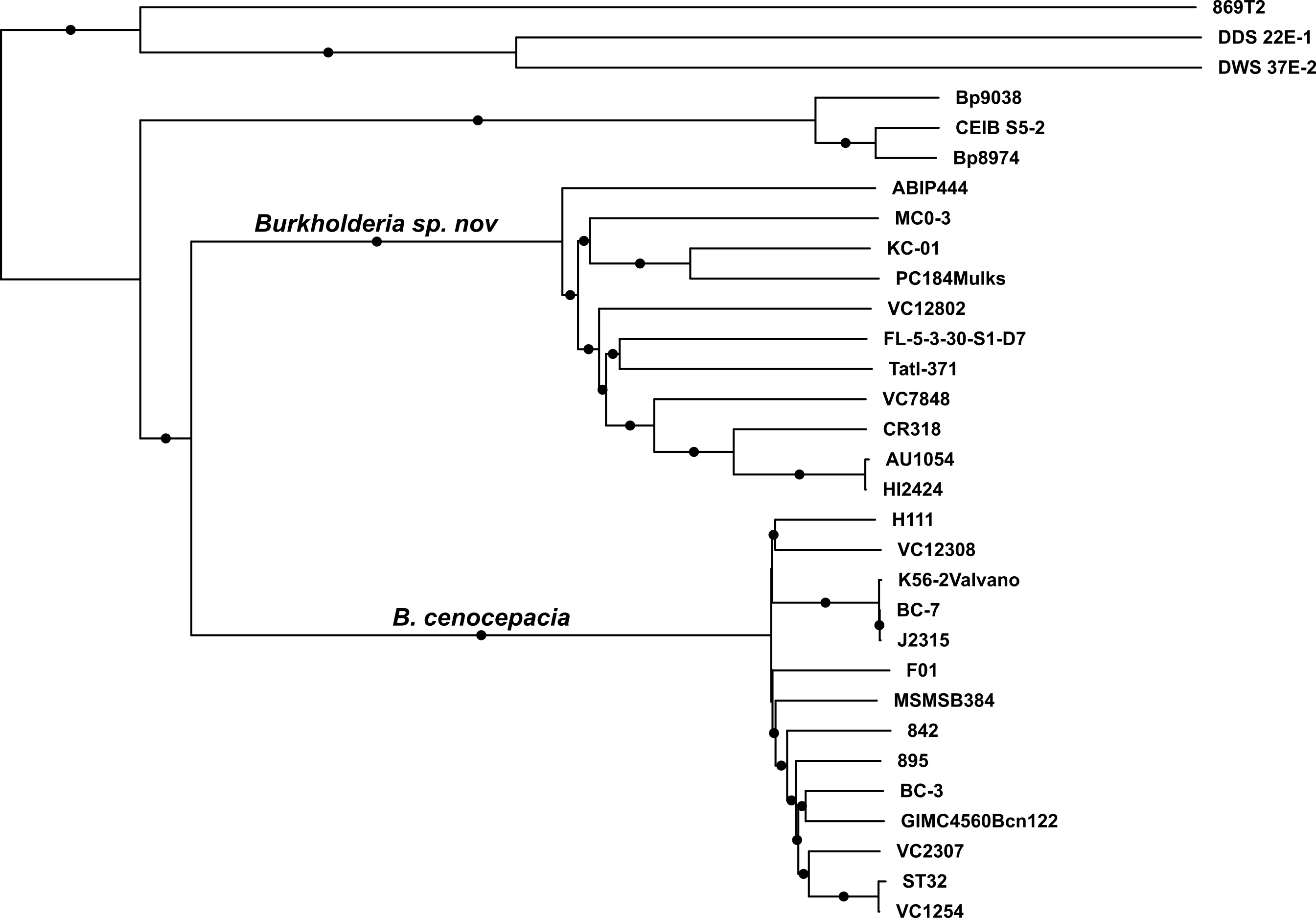

Supplement: Supplementary file 3 — Additional file 3: Figure S3. Phylogenetic tree of 31 B. cenocepacia strains. The input data is the same as for Fig. 1. The evolutionary history was inferred using the Neighbor-Joining method. The associated taxa that clustered together in > 95% of replicate trees in the bootstrap test (1000 replicates) are displayed as black dots on the tree branches. The tree is drawn to scale. The evolutionary distances were computed using the Maximum Composite Likelihood method and are in the units of the number of base substitutions per site. The analysis involved 31 nucleotide sequences. All positions with less than 95% site coverage were eliminated. That is, fewer than 5% alignment gaps, missing data, and ambiguous bases were allowed at any position. There were a total of 1,118,599 positions in the final dataset. [file 12864_2019_6186_MOESM3_ESM.png]

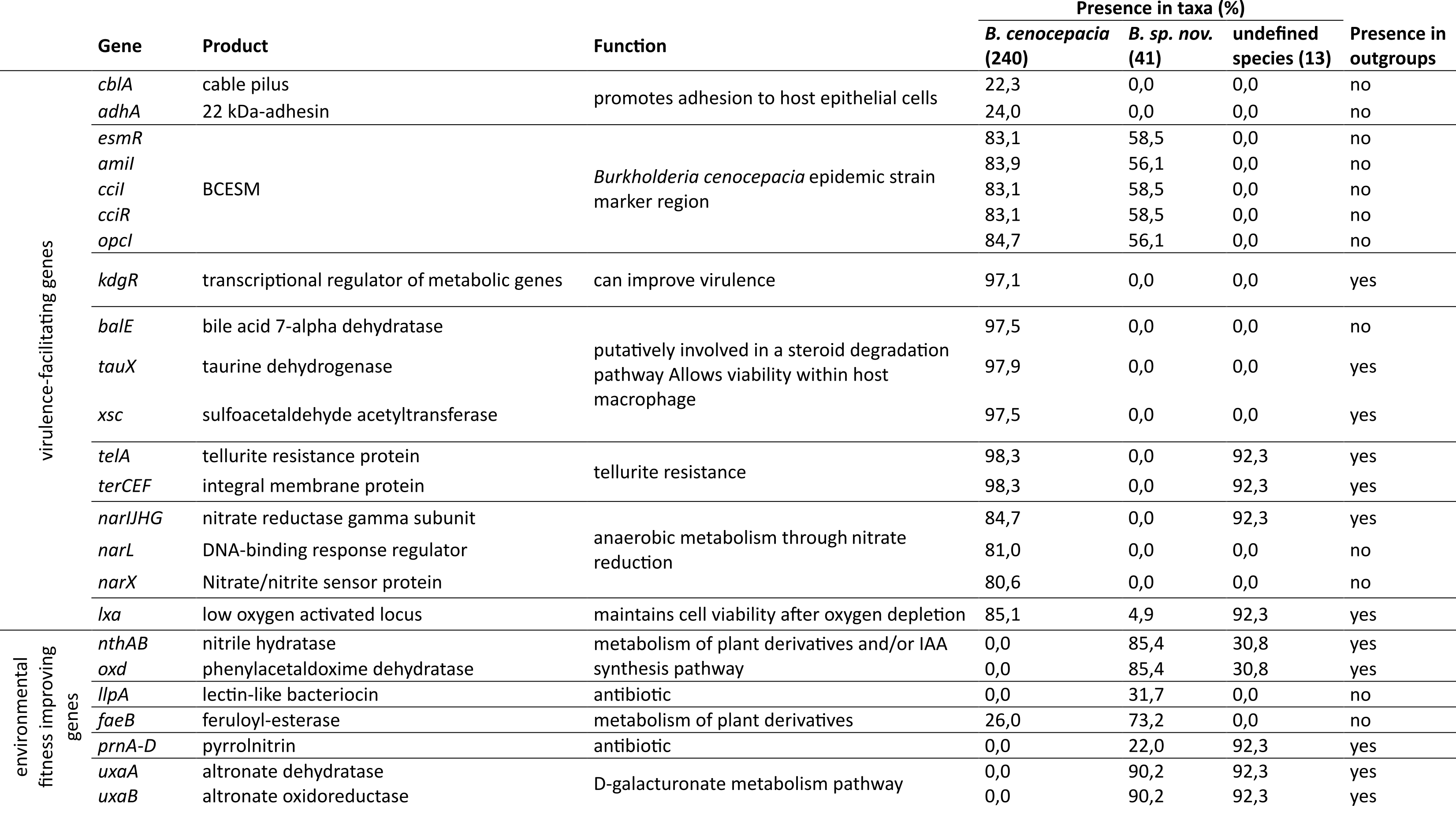

Supplement: Supplementary file 8 — Additional file 8: Table S5. Distribution across taxa of virulence facilitating and environmental fitness improving genes. For each gene, their product and their function in virulence or environmental fitness are described. The nucleotide sequence of each gene was aligned against a database of 304 B. cenocepacia genomes using BLAST. The percentage of occurrence among strains of each major taxon (B. cenocepacia, Burkholderia sp. nov. and the third undefined taxon) is given. The table also shows if a gene was found in a least one outgroup strain. [file 12864_2019_6186_MOESM8_ESM.png]
